# In vitro gentamicin release from commercially available calcium-phosphate bone substitutes influence of carrier type on duration of the release profile

**DOI:** 10.1186/1471-2474-7-18

**Published:** 2006-02-26

**Authors:** Hein P Stallmann, Chris Faber, Antonius LJJ Bronckers, Arie V Nieuw Amerongen, Paul IJM Wuisman

**Affiliations:** 1 Orthopaedic Surgery, VU Medical Center, PO Box 7057, 1007 MB Amsterdam, The Netherlands; 2 Oral Cell Biology / ACTA. vd Boechorststraat 7, 1081 BT Amsterdam, The Netherlands; 3 Oral Biochemistry / ACTA, vd Boechorststraat 7, 1081 BT Amsterdam, The Netherlands

## Abstract

**Background:**

Polymethyl-methacrylate (PMMA) beads releasing antibiotics are used extensively to treat osteomyelitis, but require surgical removal afterwards because they do not degrade.

**Methods:**

As an alternative option, this report compares the *in vitro *gentamicin release profile from clinically used, biodegradable carrier-materials: six injectable cements and six granule-types. Cement cylinders and coated granules containing 3% gentamicin were submerged in dH_2_O and placed in a 48-sample parallel drug-release system. At regular intervals (30, 90, 180 min. and then every 24 h, for 21 days), the release fluid was exchanged and the gentamicin concentration was measured. The activity of released gentamicin was tested on *Staphylococcus aureus*.

**Results:**

All combinations showed initial burst-release of active gentamicin, two cements had continuous-release (17 days). The relative release of all cements (36–85%) and granules (30–62%) was higher than previously reported for injectable PMMA-cements (up to 17%) and comparable to other biodegradable carriers. From the cements residual gentamicin could be extracted, whereas the granules released all gentamicin that had adhered to the surface.

**Conclusion:**

The high release achieved shows great promise for clinical application of these biodegradable drug-carriers. Using the appropriate combination, the required release profile (burst or sustained) may be achieved.

## Background

Osteomyelitis is a refractory condition, potentially leading to amputation or even death; treatment often requires multiple surgical interventions and local or systemic antibiotic therapy [1]. It predominantly affects both extremes of age; acute hematogenous infection occurs mainly in children and chronic osteomyelitis in the elderly [2]. Chronic osteomyelitis often requires surgical debridement and local antibiotic treatment. Disadvantages of currently used non-biodegradable polymethyl-methacrylate (PMMA) carriers include low antibiotic release by cements and the requirement of surgical removal in the case of PMMA-beads [3]. Moreover, resistant bacteria may appear on the carrier-surface during later stages of low-level antibiotic release [4]. In contrast, biodegradable antibiotic carriers may result in high release and obviate the need for removal; they are gradually replaced by ingrowing tissue [5,6]. Furthermore, secondary release of the antibiotic may occur during the degradation phase of the carrier, this could increase the antimicrobial efficacy compared to non-biodegradable carriers [7].

To our knowledge, this is the first study to describe *in vitro *antibiotic-release from twelve clinically available biodegradable carriers. The release profile of the drug-carrier combination determines the clinical efficacy for an important part. For prevention of infection, a high initial burst-release has been suggested, whereas treatment of chronic infection may require a sustained antibiotic-concentration [3,8]. The materials used, six injectable cements and six pre-shaped granule types, are all calcium-phosphate based bone-defect fillers, mainly for non-weight-bearing applications. The chemical composition of these materials is highly similar to natural bone; all consist of calcium-phosphate ceramics and are slowly replaced by ingrowing bone [9].

## Methods

### Sample production

Gentamicin-sulphate (GS) loaded cements were produced by mixing cement powder and liquid containing 30 mg GS (Biomet Merck BioMaterials, Darmstadt, Germany) per gram powder (liquid-powder-ratio according to the manufacturer). Cylinders hardened overnight (37°C) in 6 × 5 mm molds. The cements were: Biobon (Biomet Merck BioMaterials), Calcibon (Biomet Merck BioMaterials), Biofil, (DePuy CMW, Blackpool, UK), Bonesource (Stryker-Leibinger, Freiburg, Germany), Chronos Inject, (Synthes, Bettlach, Switzerland) and Norian SRS (Synthes).

One gram of granules was immersed in 2.0 mL of dH_2_O containing 30 mg GS per gram carrier-material and freeze-dried. After removal of the granules, the residual GS in the vessel was measured. The granules were: Allogran-R (Orthos, Bristol, UK), Bicalphos (Medtronic, Memphis, TN, USA), Biosorb (Science for Biomaterials, Lourdes, France), Bonesave (Stryker-Leibinger), Cerasorb (Curasan, Kleinostheim, Germany) and Vitoss (Orthovita Malvern, PA, USA).

### Release experiment

As in previous experiments [10,11], specimens were immersed in 500 μL dH_2_O, in sealed polystyrene 48-well plates (Costar, High Wycombe, UK) at room temperature on a shaking device (180 rpm). The water was replaced regularly: 30, 90 and 180 minutes on day 1, and then 24 hourly for 21 days (cements) or five days (granules) and stored at -20°C. Additional time points were used in the first 24 hours in order to detect a possible burst-release pattern. Immediately after the sample-production (control) and after the release experiment (residual) three specimens per group were finely ground and suspended in 4 mL dH_2_O containing 1 M NaCl to determine the extractable amounts of GS. The cumulative release on day 21 (cements) or five (granules) was compared with Student's *t*-test (two-tailed, p < 0.05).

### Gentamicin determination

Gentamicin concentrations were measured using an AxSym System (Abbott Laboratories, Irving, TX, USA), which allows accurate measurement of 0.30 to 30.00 μg/mL. Aliquots were therefore diluted in phosphate buffered saline (PBS); the values reported were corrected for the dilution factor. All measurements were calculated as GS quantities for transparent comparison to the amount of powder added during production. The accuracy was calculated as 2.9 % (mean error of true value) and the precision as 1.6% (coefficient of variance), it was sensitive to 0.27 μg/mL with 95% confidence [7]. The values represent the mean ± S.D. from three experiments, totalling at least nine samples per carrier-material. Cements were divided in *high release *and *low release*; the threshold was arbitrarily set at 20 mg/g or 67% release. Since all granules released <20 mg/g this division was not made for granules.

### Antimicrobial activity

A killing assay was used to ascertain gentamicin activity was undiminished by possible interaction with the calcium-phosphate. As described earlier [11], samples of 500 μL release-medium (taken after 24 hours) were freeze-dried and 10^6 ^cfu *Staphylococcus aureus *ATCC 10834 were added in 100 μL of 1 mM potassium-phosphate-buffer (pH 8.0) containing 1.0% Brain Heart Infusion. After 60 minutes incubation at 37°C, these were diluted in PBS and quantitatively cultured. The percentage of killing was calculated: [1-(cfu in sample/cfu in control)] × 100%, (>90% was considered active). Sterile cultures were conservatively calculated as 99.8% killing, which was the detection limit of the assay. Samples without GS were used as controls; samples from the first day were used as representative samples for the drug-carrier combination since these all contained sufficient amounts of gentamicin.

## Results

**Figure 1:**
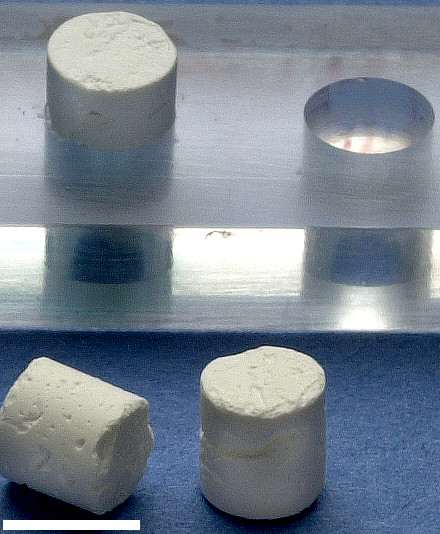
Production mold and CaP cylinders 6x5mm (A); the cylinders are slightly conical to facilitate extrusion. The scale bars are 5 mm

**Figure 2:**
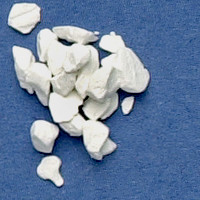
Samples of the granule types illustrate the differences in shape and macro-porosity: *Allogran*

**Figure 3:**
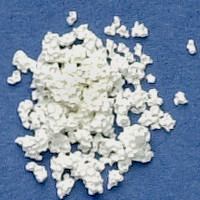
Bicalphos

**Figure 4:**
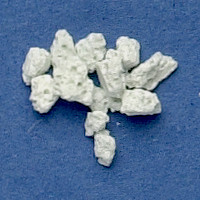
Bonesave

**Figure 5:**
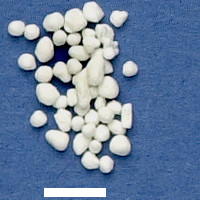
Cerasorb

**Figure 6:**
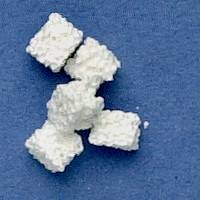
Biosorb

**Figure 7:**
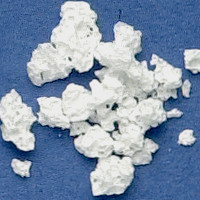
Vitoss

The cumulative release varied from 36% to 85% (cements) and 30% to 62% (granules) of the GS added during production. High killing percentages (99–100%) indicated intact antimicrobial activity (Table 1). Figure 1, 2, 3, 4, 5, 6 and 7 show the cement-cylinders, the mold and the different irregularly porous granule-types. All cements showed burst-release in the first day, two cements (*Biobon *and *Bonesource*) showed a continuous-release for 17 days. From all cements residual gentamicin could be extracted, which was highest in *Calcibon *and *Norian *(Table 1).

**Figure 8:**
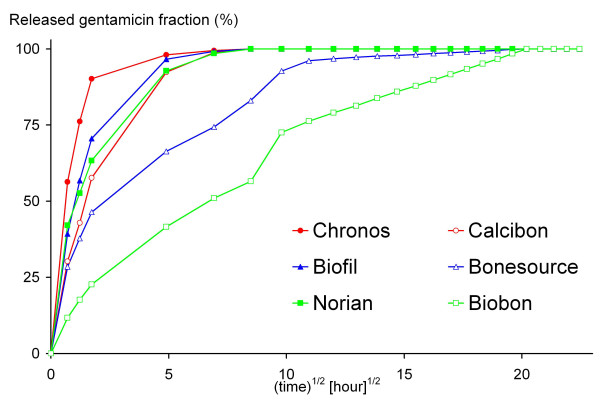
Gentamicin released from biodegradable cements. The cumulative released GS fraction as part of the total release is plotted as a function of the square root of time. Initial release was linear (R^2^ > 0.9). Data represent cumulative values from a complete fluid exchange experiment.

**Figure 9:**
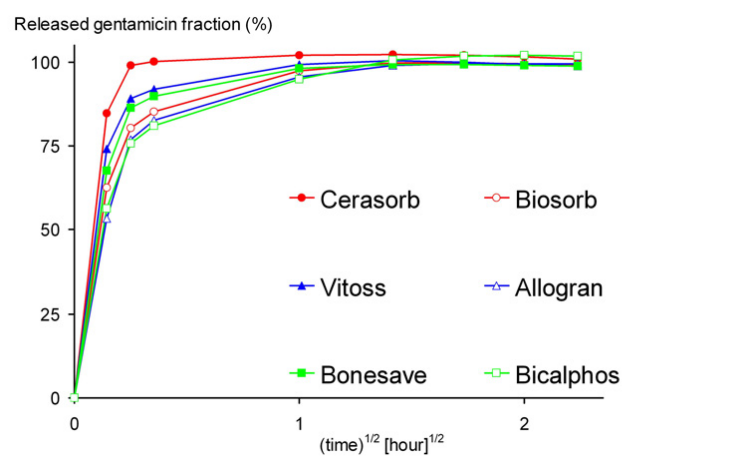
Gentamicin released from biodegradable granules. A burst release pattern was observed in all sample groups.

The *high release *cements (*Chronos, Bonesource *and *Biofil*) released more GS than *low release *cements (*Norian *and *Biobon*) and than *low release *granules *Bonesave *and *Biosorb *(p < 0.05, Table 1). The release from *Chronos *was also higher than from *low release *cement *Calcibon *(p < 0.05), the differences between Bonesource, Biofil and Calcibon were not significant. In Figure 8 the release of GS from cements is expressed as a fraction of the total cumulative release. In Figure 9 release results from the granules are plotted. The initial release, both from cements and from granules, follows square-root-of-time kinetics.

The granules all had burst-release during the first day, which was related to the amount of GS that had associated with the carrier-material during production (Table 1, *control *column). The release from the granules showed a larger variation than from cements, no significant differences between the different granule types were observed. The GS that did not associate with the granules during production was recovered from the vessels in which the samples had been freeze-dried; no residual GS was extracted from the granules.

## Discussion

A safe release profile would feature a short duration of antibiotic release after which the release should stop completely to prevent sub-inhibitory gentamicin concentrations, which could induce resistant bacteria [12]. A discrete burst after implantation eradicating the contaminating bacteria in the surgical site could be sufficient for prophylactic therapy. This would fit the burst-release seen in all carrier-materials of the current study. The continuous-release profile for 17 days seen in *Biobon *and *Bonesource *could conform well with treatment of established osteomyelitis in which a concentration ≥ 8 μg/mL for several weeks has been propagated [13,14].

Remarkably, all twelve carriers showed a higher release of active GS than reported from injectable PMMA cements. The release in the first seven days was 36–78% for cements and 30–62% for granules. Based on similar experiments both Kühn and van de Belt reported up to 17% release after seven days [14-16] Using the same conditions as the current study, Faber and coworkers reported 18% release from small sized PMMA-cement samples [17].

The GS content is a major determinant of cumulative release. This study used 3.0%, a review of current antibiotic containing PMMA cements reports a range of 2.3–3.8%. [15] Another important factor is the surface to volume ratio; our study used 6 × 5 mm cylinders (diameter × height). Compared to our study, both Kühn and van de Belt used larger sized samples, which may have influenced the total release[14,15] The importance of carrier size was demonstrated *in vivo *by Walenkamp; small PMMA-beads established a markedly higher gentamicin release (over 90%) than larger ones. [18] In contrast to biodegradable materials, PMMA-beads allow staged treatment and require surgical removal after each treatment episode.

As anticipated, the antimicrobial activity was retained; gentamicin is a heat-stable aminoglycoside and has been shown to release intact from several carrier-materials [6,19,20]. From the *cements*, not all GS could be extracted, suggesting incorporation of gentamicin into the cement. The mechanism could be by interaction of the positively charged gentamicin-base with the calcium-phosphate crystal-structure during the setting reaction [11,13]. This interaction could have been of influence on the continuous release seen in Biobon and Bonesource. Some of the (non-released) residual GS was not strongly bound to the cement matrix and could be extracted by grinding the cement samples and high salt concentrations to decrease charge dependant binding. This fraction may have been sequestered in the deeper layers, inaccessible to the release fluid. *In vivo*, it could cause prolonged release from the carrier during biodegradation, when ingrowing cells and crack formation increase the carrier-surface exposed to extracellular fluid. Carrier-surface characteristics and drug-sequestration appear to significantly influence the release profile; which could be used to tailor the therapeutic effect of drugs [14]. Favourable release properties should result in consistent safe therapeutic drug concentrations. Although all carriers used exist of calcium-phosphate salts, the chemical composition of the individual products differs. This could influence release properties by diferences in material characteristics as surface roughness, wettability and chemical reactivity of the surface [14].

The variation in total release was smaller in cements than in granules, possibly indicating that the cement mixing procedure gives a more homogeneous drug-distribution than drug-adsorption onto the irregular porous granules-surface. Both the initial burst from the cements and the complete burst-release from the granules follow square-root-of-time kinetics (Figures 8 and 9). Such kinetics would indicate that initial release from these carriers is predominantly by diffusion [13,21]. This suggests attachment of the drug both to the carrier-surface and to the internal surface of the pores. During preparation, not all GS associated with the granules, indicating that the granules' porosity and surface properties may limit the amount of GS that will attach.

This *in vitro *study is limited by the extrapolation of the results to *clinical *concentrations in bone and surrounding tissues. Many different models to analyze drug-release have been described, all optimized for certain factors that influence release including: release medium, fluid exchange volume, flow rate, and interfacial gap [22-24]. In this model testing conditions were rigidly kept constant for all groups, but not all physiological parameters were mimicked: clearance of the drug by blood flow, buffered pH and ionic content, and the high concentration in the carrier-bone interface were not included in this model [22]. This experiment used complete exchange of small volumes of water as the release medium, allowing comparison with our earlier experiments that reported dose-dependent release of antimicrobial agents from PMMA and biodegradable carriers [10,11]. In animal experiments, we successfully used calcium-phosphate cement loaded with gentamicin to prevent infection in a well characterized rabbit model of osteomyelitis. Bone cultures, the gold standard in osteomyelitis detection, showed a significant reduction in pathogenic bacteria [25]. This stresses the potential therapeutic options of such composites [26]. As a technical advantage, the current set-up will test several combinations in one experimental run, since it accommodates a large number of isolated samples.

The strength of *in vitro *release studies of this kind lies in a qualitative comparison of different carrier materials, allowing the identification of the most suitable drug-carrier composites. Although recognizing the limitations of *in vitro *models, a positive correlation between *in vivo *and *in vitro *results has been reported for gentamicin-containing biodegradable implants [27,28]. This study presents several high release carriers. Possible advantages of cements include high and continuous release and easy injection into the relevant anatomic site. We have succesfully used antibiotic containing cement in small animal models [26]. On the other hand, the granules completely released all gentamicin present, markedly decreasing the risk of inducing antimicrobial resistance.

Prolonged in-hospital systemic antibiotic treatment of osteomyelitis implies a heavy burden for both patient and healthcare organisation. The large number of currently available non-biodegradable antibiotic carriers underscores the demand for sound local antibiotic treatment options. In contrast, only few approved biodegradable antibiotic delivery devices are currently available. This emphasizes the clinical potential of biodegradable antibiotic carriers and the need for comparative studies. By using materials approved for implantation in bone, the gap between pre-clinical and clinical studies may perhaps be narrowed, thus allowing early adaptation and implementation of these devices.

## Conclusion

For patients requiring surgery for bone defects, the burst-release pattern observed in all carriers, may offer clinical applications for the prevention of osteomyelitis. The prolonged release-profile from two of the cements might be an effective option in the concept of treating osteomyelitis [8,29]. Since all carrier-materials are commercially available, these results may readily be extended to *in vivo *studies to determine the optimal combination for different clinical situations. The presented results support the ongoing clinical exploration of antibiotic containing biodegradable drug-delivery systems [5,19,26].

## Competing interests

This study was made possible by Senter Grant TSGE 1044 and AM-Pharma BV (Bunnik, the Netherlands).

## Authors' contributions

HS and CF were the primary researchers, AB supervised the experiments, worked on data processing and writing. PW and AA were the directors of the study. All authors read and approved the final manuscript.

## Pre-publication history

The pre-publication history for this paper can be accessed here:



## Figures and Tables

**Table 1:** All carriers combined high cumulative release with intact antimicrobial activity, only the cements had residual GS after release. The total release is also given as percentage of the 30 mg/g that was added during production. (^a^)Total release from three *high release *cements (*Biofil*, *Bonesource *and *Chronos*) was significantly higher than from *low release *cements *Biobon *and *Norian *and than *low release *granules *Bonesave *and *Biosorb*. (^b^)The release from *Chronos *was also higher than from *low release *cement Calcibon (all p < 0.05). Only two cements showed continuous release (*Biobon *and *Bonesource *for 17 days). For comparison, available gentamicin extracted from samples ground immediately after production is shown in the *control *column. Values are means ± SD of at least three experiments in triplicate.

**Carrier**	Gentamicin (mg/g)				**Killing**
		
	**cumulative release **	**(%)**	**residual **	**control**	**(%)**
*Cements*					

Biobon	14.0 ± 3.6	46.7	2.1 ± 2.1	17.1 ± 0.0	99.8
Biofil	23.2 ± 4.0	77.3^a^	0.9 ± 0.3	21.3 ± 3.9	99.7
Bonesource	25.3 ± 2.5	84.3^a^	1.8 ± 2.0	23.4 ± 4.5	99.8
Calcibon	16.1 ± 5.9	53.7	5.8 ± 3.8	21.2 ± 0.5	99.8
Chronos	25.6 ± 3.3	85.3^a,b^	0.5 ± 0.3	22.0 ± 5.5	99.1
Norian	10.8 ± 3.8	36.0	6.4 ± 0.0	16.9 ± 8.4	99.8
*Granules*					

Allogran	15.2 ± 9.6	50.7	0.0	16.5 ± 10.0	99.8
Bicalphos	13.4 ± 8.2	44.7	0.0	15.3 ± 9.9	99.8
Biosorb	9.6 ± 2.7	32.0	0.0	11.5 ± 6.0	99.8
Bonesave	9.1 ± 5.5	30.3	0.0	10.0 ± 4.6	99.7
Cerasorb	10.8 ± 7.1	36.0	0.0	11.1 ± 7.1	99.8
Vitoss	18.6 ± 8.3	62.0	0.0	18.6 ± 10.1	99.4
